# Study on the Effectiveness of Two Biopolymer Coagulants on Turbidity and Chemical Oxygen Demand Removal in Urban Wastewater

**DOI:** 10.3390/polym15010037

**Published:** 2022-12-22

**Authors:** Miguel Mauricio Aguilera Flores, Gloria Itzel Valdivia Cabral, Nahum Andrés Medellín Castillo, Verónica Ávila Vázquez, Omar Sánchez Mata, Jésica García Torres

**Affiliations:** 1 Interdisciplinary Professional Unit of Engineering, Campus Zacatecas, Instituto Politécnico Nacional, Blvd. del Bote 202, Cerro del Gato Ejido la Escondida, Col. Ciudad Administrativa, Zacatecas 98160, Zac, Mexico; 2 Graduate Studies and Research Center, Faculty of Engineering, Autonomous University of San Luis Potosi, Av. Manuel Nava No. 8, Col. Zona Universitaria Poniente, San Luis Potosí 78290, SLP, Mexico

**Keywords:** biopolymer coagulants, coagulation, optimal dose, *Opuntia robusta*, *Uncaria tomentosa*, urban wastewater treatment, water quality

## Abstract

The present study investigated the effectiveness of two biopolymer coagulants on turbidity and chemical oxygen demand removal in urban wastewater. The biopolymers were produced from vegetal biomass using the mucilage extracted from *Opuntia robusta* cladodes, and *Uncaria tomentosa* leaves. *Opuntia robusta* is an abundant species in Mexico, which is not edible. *Uncaria tomentosa* is an exotic invasive species in Mexico and other countries, which negatively affects the ecosystems where it is introduced. A combined experimental design of mixture–process was selected to evaluate the effectiveness of both biopolymer coagulants regarding aluminum sulfate (conventional chemical coagulant). Results showed turbidity and chemical oxygen demand removal efficiencies of 42.3% and 69.6% for *Opuntia robusta* and 17.2% and 39.4% for *Uncaria tomentosa* biopolymer coagulant, respectively, at a dose of 200 mg/L. Furthermore, optimum conditions from the experimental design to reach the maximum turbidity and chemical oxygen demand removal were obtained at an *Opuntia robusta* biopolymer coagulant concentration of 10 mg/L, showing removal efficiencies of 68.7 ± 1.7% and 86.1 ± 1.4%, respectively. These results support using *Opuntia robusta* as an alternative biopolymer coagulant in urban wastewater treatment.

## 1. Introduction

Water is a resource essential for life. However, freshwater resources are dwindling at an alarming rate. Growing freshwater scarcity is now one of the most significant challenges for sustainable development [1]. Over two billion people live in water-stressed countries, exacerbating in some regions due to climate change and population growth. Globally, at least two billion people use a drinking water source contaminated with feces, causing 485,000 diarrheal deaths yearly. Microbiologically contaminated drinking water can transmit diseases such as cholera, diarrhea, dysentery, polio, and typhoid [2].

The United Nations adopted the Sustainable Development Goals (SDG) in 2015 as a universal call to action to end poverty, protect the planet, and ensure that by 2030 all people enjoy peace and prosperity. One of the targets of SDG 6: “Cleaner water and sanitation” is to “By 2030, improve water quality by reducing pollution, eliminating dumping and minimizing release of hazardous chemicals and materials, halving the proportion of untreated wastewater and substantially increasing recycling and safe reuse globally” [3]. Safe and sufficient water facilitates the practice of hygiene, a key measure to prevent diarrheal diseases, acute respiratory infections, and numerous neglected tropical diseases [2]. Therefore, affordable wastewater treatment alternatives to reduce untreated wastewater and increase reclaimed water-safe reuse remains an area of opportunity.

A wide range of physical, chemical, and biological technologies have been developed to treat water and wastewater in response to the severe problem of water scarcity. Coagulation is a chemical technology frequently employed in primary and advanced water and wastewater treatment [4]. This technology is an efficient process of removing impurities (suspended particles and colloids) in water, combining small particles into larger aggregates (flocs), and adsorbing dissolved organic matter onto particulate aggregates, which can be removed in subsequent solid–liquid separation processes [5].

Coagulation is based on the use of chemical coagulants. Aluminum salts (aluminum sulfate), hydrated lime, iron salts (ferric chloride and ferric sulfate), magnesium carbonate, and polymers (aluminum chlorohydrate, polyaluminum chloride, polyaluminum sulfate, and polyferric sulfate) are the chemical compounds commonly used to remove pollutants from wastewater [6]. However, their application implicates adverse environmental impacts, which include increasing the corrosion rate of metallic utilities, changing the water pH, limiting root elongation, inhibiting seed germination, and generating excessive chemical sludge that requires adequate treatment. Likewise, chemical coagulants are nonbiodegradable compounds, and their presence in water could cause impact human health (Alzheimer’s disease, central nervous system failure, dementia, and severe trembling) when consumed and accumulated in body cells [6,7].

Biocoagulants can be an alternative to minimize the environmental pollution and health risks caused by chemical coagulants [6]. Therefore, the search for biocoagulants is an exploration area in the challenge of sustainable development. These materials are based on biopolymers, such as cellulose, mucilage, natural gums, and starch. They are derivatives from microorganisms, plant, and animal bases [6,7,8]. Adsorption, polymer bridging, and charge neutralization are the three main biocoagulation mechanisms than have been described for these biomaterials [6]. The main advantages of biopolymers are biodegradability, nontoxicity, renewability, and relative cost-effectiveness [6,8,9].

Some biocoagulants employed in water and wastewater treatment are *Penicillium* sp. and *Trichoderma* sp. spores (microorganism-based), reducing 84% of turbidity in domestic wastewater. The result was alum-like efficiency [10]. *Moringa oleifera* seed (plant-based) was used to treat aquaculture wastewater. A high coagulation efficiency was achieved (>95%), showing a better performance than aluminum sulfate at a low dosage of 10 mg/L and pH 6.9–7.5 [11]. Likewise, a turbidity removal efficiency of >85% was obtained in drinking water treatment [12]. Combining crab shell biocoagulant (animal-based) and alum reduced the low-, medium-, and high-turbidity of drinking water by 74.8%, 96.7%, and 98.2%, respectively. These results were higher than those using only alum coagulant [13].

Research has recently focused on plant-based and marine biomass-based biopolymer coagulants [4,14,15]. Chitosan, moringa, and tannin are commercially available biocoagulants [16]. Other reported biocoagulants have not been commercialized, remaining in lab-scale trials, or employed in small-scale (case-specific applications) [4]. Therefore, research to further improve biopolymer coagulants’ ability and increase confidence in their industrial application remains an area of opportunity.

The interest in producing biopolymer coagulants increases because it is possible to obtain these compounds from invasive species, biomass waste, or species with a high population density that have no use. This study proposes using *Opuntia robusta* cladodes and *Uncaria tomentosa* leaves to produce biopolymer coagulants and remove pollutants (turbidity and chemical oxygen demand) in urban wastewater.

Cactaceae is one of the most cultivated and used plant families as an essential source of income for large populations of the American continent. In Mexico, more than 150 species of cacti are used by local communities [17]. *Opuntia* is a genus of plants in the Cactaceae family, characterized by its high potential for biomass production, and is widely used for human consumption. *Opuntia* cladodes are used in folk medicine to treat gastric mucosa diseases, reporting that the mucilage content is probably involved [18]. This plant has a high concentration of polysaccharides, principally mucilage. This substance contains arabinose, galactose, galacturonic acid, rhamnose, and xylose. These components are considered soluble fibers and can form gels in water. For these reasons, *Opuntia* mucilage has been used in the bioremediation of wastewater [18]. *Opuntia robusta* is an abundant species in Mexico; it is found in the wild and is not edible. Hence, the mucilage of this plant could be used as a biopolymer coagulant and yield a value-added product.

*Uncaria tomentosa* is a plant native to the Amazon region and distributed on various continents. This species is used widely in popular medicine and the pharmaceutical industry, since it exhibits anti-inflammatory activity [19]. *Uncaria tomentosa* has become a significant invader of cultivated orchards, riparian corridors, natural forest remnants, and disturbed areas, such as roadsides and urban spaces. Its vigorous growth allows it to sprawl over other vegetation, and through shading and weight, it can even kill large canopy trees. Likewise, it inhibits the growth and seed germination of native understory vegetation, including native grasses, herbs, and seedlings of shrubs and trees [20]. The chemical composition of *Uncaria tomentosa* involves carotenoids, flavonoids, mineral salts, organic acids, and mucilage [21]. Therefore, these properties could be exploited to produce a biopolymer coagulant and have biological control over this invasive species.

It is worth mentioning that species of the *Opuntia* genus have been used to produce biocoagulants. However, there is no information in the consulted literature where either *Opuntia robusta* or *Uncaria tomentosa* have been used as a biopolymer coagulant, so their study represents an area of opportunity in their use for this purpose. In this context, this manuscript aims to evaluate the effectiveness of two biopolymer coagulants from *Opuntia robusta* and *Uncaria tomentosa* mucilage for removing turbidity and chemical oxygen demand in urban wastewater. Studies of the effect at different concentrations using the two biopolymer coagulants and their combination with aluminum sulfate were performed under an experimental design. This work presents a solution alternative to water scarcity, proposing the use of biocoagulants in the treatment of urban wastewater, contributing to the target of SDG 6: “Cleaner water and sanitation”.

## 2. Materials and Methods

### 2.1. Raw Material

*Uncaria tomentosa* leaves were collected in the wild in Apulco (Zacatecas, Mexico), located at latitude 21°23′25″ N and longitude 102°41′04″ W. Leaves of approximately 5 cm length were selected and cut using pruning pliers. The collected samples were placed inside plastic bags to be transported to the laboratory.

*Opuntia robusta* cladodes were collected in the wild in Trancoso (Zacatecas, Mexico), located at latitude 22°44′25″ N and longitude 102°22′27″ W. Cladodes of more than 15 cm in diameter were selected and cut with a knife. The thorns were removed, and the cladodes without thorns were placed inside plastic bags. Later they were transported to the laboratory.

Both species were authenticated as *Uncaria tomentosa* and *Opuntia robusta* based on their morphology. Approximately 1.2 kg of *Uncaria tomentosa* leaves and 1 kg of *Opuntia robusta* cladodes without thorns were collected to produce the biopolymer coagulants.

The urban wastewater sample was used as a case study to evaluate the effectiveness of the biopolymer coagulants. Approximately 15 L of wastewater sample was collected at the discharge point of urban wastewater in Zacatecas (Zacatecas, Mexico).

Aluminum sulfate (Mca. Meyer reagent grade) was used as the conventional chemical coagulant. A commercial flocculant (anionic polymer) was used in the jar tests. This product was provided by a Mexican company.

### 2.2. Biopolymer Coagulants Production and Characterization

The mucilage extracted from *Uncaria tomentosa* and *Opuntia robusta* was used as a biopolymer coagulant. The mucilage extraction was performed following the methodology proposed by Sáenz et al. [22]. A pretreatment for each sample was performed. *Uncaria tomentosa* leaves were washed with tap water. Then, they were placed in a beaker with deionized water at a 1:2 *w*/*v* ratio for 24 h. The bark of the *Opuntia robusta* cladodes without thorns was removed using a knife. The obtained material was washed with tap water and cut into small proportions.

After these pretreatments, each material was mixed in a domestic blender using distilled water at a 1:2 *w*/*v* ratio, and the mixture was placed in an oven at 50 °C for 60 min. After, it was centrifuged at 3500 rpm for 10 min. Ethanol was added to the supernatant recovered in the centrifugation at a 1:4 *v*/*v* ratio. This mixture was kept at 4 °C for 24 h. Later, the mixture was vacuum filtered, and the solid particles retained in the filter medium were dried at room temperature for 24 h. Once the material was dry, it was crushed in a mortar. The product was stored in a glass bottle with a lid at 5 °C until its use.

Yields of approximately 3 and 15 g of biopolymer coagulant per kg wet weight of *Uncaria tomentosa* leaves and *Opuntia robusta* cladodes were obtained, respectively.

The biopolymer coagulants characterization included the pH and moisture content measures and infrared analysis. The pH was determined using a LAQUAact PH110 Potentiometer from Horiba Scientific from a 1% solution of each biopolymer coagulant prepared with distilled water. The moisture content was determined by direct measurement using an OHAUS Moisture Analyzer MB45 and 1 g of each biopolymer coagulant.

Spectra of each biopolymer coagulant were obtained by Fourier transform infrared (FTIR) spectroscopy to identify functional groups and predict the coagulation mechanisms. The spectra were collected in 32 scans at 4 cm^−1^ in the mid-IR range 4000–400 cm^−1^ with automatic signal gain and rationed against a background spectrum recorded from the clean empty cell at 25 °C. Spectral data analysis was performed using the OPUS 3.0 data collection software program.

### 2.3. Experimental Design and Jar Test

The effectiveness of the biopolymer coagulants was evaluated under a combined experimental design of mixture–process. This design consisted of a mixture of three components: *Opuntia robusta* biopolymer coagulant, *Uncaria tomentosa* biopolymer coagulant, and aluminum sulfate. The component values were interpreted as proportions of the coagulant dose to be used. The levels ranged from 0 to 1 for each component. The sum of the components’ proportions in the mixture was 1 for each trial. The coagulant dose was the numeric factor to be studied. This factor ranged from 200 to 800 mg/L. Thirty trials resulted from the experimental design (Table 1). The trials were performed at the laboratory in random order. The percentages of turbidity and chemical oxygen demand removal were the response to be analyzed.

The coagulation tests were performed using jar floc test equipment (Flocculator SW6, Bibby Stuart, Armfield). The urban wastewater sample was mixed well before undertaking the jar test. The jar test was carried out at room temperature using 500 mL of urban wastewater in each trial. The jar test involved three steps: (1) rapid mixing at 150 rpm for 5 min with coagulant addition according to the trial number shown in Table 1, (2) slow mixing at 50 rpm for 30 min with 0.5 mL flocculant addition prepared at a concentration of 1 mg/L, and (3) sedimentation for 60 min. Later, a volume of liquor supernatant (~40 mL) was pulled at 5 cm from the sample surface for conducting the final water physicochemical characterization. Each test was performed in duplicate, reporting the average value.

### 2.4. Water Physicochemical Characterization

Temperature, pH, electrical conductivity, turbidity, and chemical oxygen demand were the physicochemical parameters analyzed in the urban wastewater samples before and after the jar test. The temperature was measured using a Mercury thermometer, the pH with a LAQUAact PH110 Potentiometer (Horiba Scientific, Kyoto, Japan), the electrical conductivity with a conductivity meter with an RS-232 Cable (Eutech Instruments Thermo Scientific, Singapur, Singapore), the turbidity with a TB200TM Portable Turbidimeter (Orbeco-Hellige, Inc., Florida, USA), and the chemical oxygen demand using the method of the Mexican Standard NMX-AA-030/2-SCFI-2011 [23]. The percentages of turbidity and chemical oxygen demand removal were calculated by Equation (1). These results were used as the responses in the experimental design.

(1) $$\mathrm{Removal}\;\left(\%\right)=100\times \left(X_{\mathrm{initial}}-X_{\mathrm{final}}\right)/X_{\mathrm{initial}}$$

X_initial_ and X_final_ are the turbidity or chemical oxygen demand values before and after the coagulation treatment, respectively. The results were expressed as the mean of two measurements. Likewise, the temperature, pH, and electrical conductivity were analyzed to identify a possible variation of these parameters when the coagulation treatment was performed.

### 2.5. Statistical Analysis

Statistical analyses were performed using Design-Expert^®^ Version 12 Software (Trial version) (Stat-Ease, Inc., Minneapolis, MN, USA). Analysis of variance ANOVA considered a combined special cubic × linear model, determining the significance of the model and the lack of fit at *p*-value < 0.05. Likewise, the final model equations in terms of the mixture’s components and doses and 3D surface plots were shown. Finally, optimization analysis was performed using the following criteria: the maximum amount of the biopolymer coagulants, the minimum amount of aluminum sulfate, a dose range of 10 to 1000 mg/L, and the final model equations.

## 3. Results and Discussion

### 3.1. Characterization of Produced Biopolymer Coagulants

*Opuntia robusta* biopolymer coagulant showed a pH of 3.77 and a moisture content of 9.96%. *Uncaria tomentosa* biopolymer coagulant showed values of 5.38 and 7.23%, respectively. These results are associated with the chemical structure. *Opuntia robusta* contains more variety and a higher quantity of carbohydrates than *Uncaria tomentosa* [18,21]. Therefore, *Opuntia robusta* shows lower pH and higher humidity.

Both biopolymer coagulants showed values of acid pH. This condition could acidify water pH once that is treated by coagulation. However, authors have reported that the biocoagulants are not sensitive to pH ranges nor do they significantly affect water pH [24]. Therefore, it is expected that water pH will not change drastically when any of the biopolymer coagulants are used during the treatment.

One of the components of the mucilage of *Opuntia* spp. is galacturonic acid. This acid has been classified as an anionic coagulant and is considered non-ionic in pH ranges of 6.5–8.5 or higher. Two coagulation mechanisms are associated with this compound. The first one is a polymeric molecule with a charge which performs the coagulation mechanism [6,25]. The second is a non-ionic polymer that provides a H+ bridge to adsorb colloidal particles [26].

**Figure 1:**
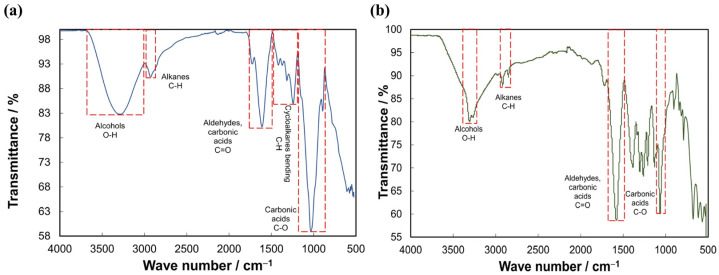
FTIR spectra of biopolymer coagulants: (**a**) *Opuntia robusta*, (**b**) *Uncaria tomentosa*.

Figure 1 shows the FTIR spectra of *Uncaria tomentosa* and *Opuntia robusta* biopolymer coagulants and the functional groups identified using FTIR conversion tables [26,27].

Other authors reported the characterization of the *Opuntia robusta* mucilage by FTIR. They observed a peak in the range 2850–2970 cm^−1^ associated with the stretching of the C–H bonds of the pyranose groups or C–H groups of the methyl ester of the galacturonic acid [28]. This stretching range of C–H bonds is observed in Figure 1a. Therefore, the presence of galacturonic acid in the *Opuntia robusta* mucilage is the main factor for performing a coagulation process of the suspended and colloidal material in urban wastewater.

On the other hand, FTIR spectra of *Uncaria tomentosa* mucilage have not been reported. The stretching range of C–H bonds is also observed in Figure 1b. Hence, this result could also be associated with galacturonic acid in the *Uncaria tomentosa* mucilage.

Infrared analysis results allowed the identification of functional groups associated with galacturonic acid in both biopolymer coagulants. This compound is the main factor, allowing coagulation mechanisms such as adsorption, charge neutralization, polymer bridging, and electrostatic patching [6]. Therefore, these species could be exploited as raw materials, producing biopolymer coagulants and bioremediating wastewater.

### 3.2. Physicochemical Parameters of Urban Wastewater before and after Coagulation Treatment

Table 2 shows the physicochemical parameters of the urban wastewater before coagulation treatment.

The temperature and pH values of the urban wastewater are within the water quality parameters established by the World Health Organization (WHO) (Table 2). Although the pH value could be slightly modified by using aluminum sulfate or both biopolymer coagulants, it would be expected that the pH will not be out of range when the urban wastewater is subjected to the coagulation treatment.

On the other hand, the electrical conductivity value of urban wastewater is up to 8.4 times higher than the lower limit stipulated by the United States Environmental Protection Agency (Table 2). This parameter is referred to as the amount of dissolved ionic components (total salts or salinity) in water [32]. Values outside this range indicate that the water is not acceptable for certain fish or macroinvertebrates [30]. Hence, this wastewater must be treated before being discharged or reused. Furthermore, an increase in this value during coagulation treatment by using chemical or biopolymer coagulants could cause a health risk to direct discharge into water bodies.

The turbidity value of urban wastewater is 11.2 times higher than the water quality value established by the WHO (Table 2). Likewise, the Mexican standard stipulates a limit value of 4 NTU in water for human use and consumption [33]. Therefore, the urban wastewater exceeds 14 times the quality parameter. Turbidity can be generated by organic particulates that harbor microorganisms and stimulate bacteria’s growth. Thus, high turbidity in water increases the possibility of waterborne diseases, representing health risks to effluent users [33].

Furthermore, excessive turbidity also makes the sight of the receiving water bodies, where the effluent was discharged, unpleasant for full-contact recreation. It increases treatment costs due to problems caused by the filtration and disinfection processes [34]. Consequently, this wastewater must be treated before being discharged or reused to avoid health risks from direct discharge into water bodies. Treatment must be based on coagulation since a sedimentation process would be inefficient, and a filtration process could be affected by fouling due to the presence of suspended particles and colloids (high turbidity) (Table 2).

The chemical oxygen demand value of urban wastewater is up 2.7 times higher than the lower limit stipulated by the Mexican standard (Table 2). This parameter measures the susceptibility to oxidation of the organic and inorganic materials in water bodies and effluents from wastewater treatment plants. High chemical oxygen demand concentrations lead to oxygen consumption, depleting it and causing alterations in aquatic ecosystems [31]. Therefore, it would be expected that the COD-causing substances would be removed by coagulation treatment.

Table 3 shows the physicochemical parameters of the water after coagulation treatment.

The highest temperature changes were ± 0.5 °C after the coagulation treatment (Table 3). These changes are not significant since they are within the water quality parameter established by the WHO (<25 °C) [29]. This parameter does not significantly affect the coagulation process since it has been reported that biopolymer coagulants are effective in cold and warm water [24].

According to the data detailed in Table 3, the water pH is slightly acidified after coagulation treatment. The highest pH changes (>−1.0) occurred when the aluminum sulfate was used alone or mixed with a proportion of 0.66 and a dose of 800 mg/L. The lowest changes in pH (<−0.3) occurred when the biopolymer coagulants were used alone or mixed between them. Hence, *Uncaria tomentosa* and *Opuntia robusta* biopolymer coagulants do not significantly affect the water pH despite the acid pH values of both biocoagulants shown in Section 3.1. Some authors have reported that the biopolymer coagulants are not sensitive or modify the water pH [24]. Therefore, this condition favors using these coagulants in water and wastewater treatment. Likewise, it should be noted that the final pH values are not outside the limits established by the WHO (6.5–8.5) [29] in any of the cases. Thus, it would facilitate an escalation of the treatment without acidifying or basifying the water to treat.

Electrical conductivity values in water after coagulation treatment increased grossly for all the cases (Table 3), mainly at a dose of 800 mg/L, since this parameter increased as the dose of coagulants grew, mainly with the presence of aluminum sulfate. This increase is associated with dissolved ions provided by the coagulants. The aluminum sulfate has a higher dissociation capacity than biopolymer coagulants, producing ions that raise the electrical conductivity values [32,35]. Therefore, this parameter must be controlled before treated water discharge, requiring refining processes such as ion exchange, adsorption, or filtration.

Although applying these refining treatments could significantly increase the wastewater treatment cost, the high electrical conductivity could also be associated with the high concentrations of biopolymer coagulants and aluminum sulfate [32]. Hence, an optimization analysis is necessary to identify the optimal doses for each coagulant. If the coagulant concentration is low, the formation of flocs is complicated due to the minimal possibility of collision between particles. Instead, if the coagulant concentration is high, the flocs are not formed properly, causing high turbidity and electrical conductivity due to the presence of dissolved particles and ions [36]. This condition could be solved by using the optimal dose of coagulant. Thus, it would no longer be necessary to resort to refining processes after the coagulation treatment to reduce the high electrical conductivity. For this reason, this study performed an optimization analysis, which is shown in Section 3.3.

The percentages of turbidity and chemical oxygen demand removal (Table 3) were used as the responses for the analysis and discussion of the results. However, it can be noted that the highest turbidity and chemical oxygen demand removal efficiency of 92.2% and 97.2%, respectively (trial 20), was reached with aluminum sulfate alone at a dose of 200 mg/L. Subsequently, trials 22 and 1 showed high removal efficiencies with values of 88.62% and 88.51% in turbidity and 92.64% and 87.15% in chemical oxygen demand at aluminum sulfate doses of 500 mg/L and 800 mg/L, respectively. It can be noted that the aluminum sulfate efficiency decreases when the coagulant dose increases.

Efficiencies of turbidity and chemical oxygen demand removal of 17.2% and 39.4% (Trial 16) and 42.3% and 69.6% (Trial 18) were obtained for *Uncaria tomentosa* and *Opuntia robusta*, respectively, when the biopolymer coagulants were used alone in coagulation treatment with a coagulant dose of 200 mg/L. The removal efficiencies were slightly decreased when the doses increased to 500 mg/L (trials 23 and 22). However, higher turbidity and chemical oxygen demand levels (zero removal efficiencies) were conferred at 800 mg/L (trials 17 and 19), as in the case of aluminum sulfate.

Trials 3, 13, 17, 19, and 27 showed zero values because the used coagulant doses were above their optimal dosage. Therefore, the final values of turbidity and chemical oxygen demand increased. These results are associated with particle destabilization and the coagulant itself. They cause more suspended particles and colloids (turbidity) and oxidizable organic matter (chemical oxygen demand) [36,37].

Efficiencies of turbidity and chemical oxygen demand removal of 88.1% and 83.0% were achieved, respectively, at a coagulant dose of 800 mg/L and a mixture proportion of 0.66 *Opuntia robusta* biopolymer coagulant and 0.33 aluminum sulfate (Trial 7). Moreover, removal efficiencies of 58.9% and 72.7% were achieved, respectively, at a coagulant dose of 200 mg/L and a mixture proportion of 0.66 *Uncaria tomentosa* biopolymer coagulant and 0.33 aluminum sulfate (Trial 4).

On the one hand, these results show that *Opuntia robusta* biopolymer coagulant has a higher potential than *Uncaria tomentosa* biopolymer coagulant. Some authors have reported a working pH range of 6.5–8.5 for *Opuntia* spp. mucilage coagulant [25]. The urban wastewater pH was 7.71 ± 0.02 (Table 2). Therefore, *Opuntia robusta* biopolymer coagulant was within the working pH range. This condition could influence the results showing *Opuntia robusta* biopolymer coagulant with better efficiencies than *Uncaria tomentosa*. The working pH range for *Uncaria tomentosa* mucilage coagulant has not been reported in the literature. Hence, this opens an opportunity area for optimizing the working pH range for *Uncaria tomentosa* biopolymer coagulant and improving its efficiency in removing pollutants from wastewater [6].

On the other hand, although the potential of *Opuntia robusta* and *Uncaria tomentosa* biopolymer coagulants is lower than aluminum sulfate, their efficiency is improved when used as a coagulant aid mixed with aluminum sulfate. These results indicate that using these biopolymer coagulants could be feasible in dual systems when mixed with aluminum sulfate. Therefore, an optimization analysis was performed considering the highest removal of the two water quality parameters (turbidity and chemical oxygen demand), the maximum amount of biopolymer coagulants, and the minimum amount of aluminum sulfate.

### 3.3. ANOVA and Optimization Analysis Results

Table 4 shows the analysis of variance ANOVA, considering the percentage of turbidity removal as the response. It can be noted that the model is statistically significant (*p*-value < 0.05), and the lack of fit is not statistically significant (*p*-value > 0.05). Hence, a combined special cubic x linear model is appropriate to describe the observed data. The significant effects are the linear mixture of the three coagulants, the double interactions between *Opuntia robusta* biopolymer coagulant and aluminum sulfate (A × C), and *Opuntia robusta* biopolymer coagulant and doses (A × D). Therefore, the final equation in terms of components and factors for this response is described by Equation (2). A, B, and C are the proportions in the mixture of *Opuntia robusta* biopolymer coagulant, *Uncaria tomentosa* biopolymer coagulant, and aluminum sulfate, respectively, and D is coagulant doses.

(2) Turbidity removal (%)=71.39×A+13.15×B+83.10×C−112.35×A×C−0.09×A×D

Table 5 shows the analysis of variance ANOVA, considering the percentage of chemical oxygen demand removal as the response. As in the ANOVA for turbidity, it can be noted that the model is statistically significant (*p*-value < 0.05), and the lack of fit is not statistically significant (*p*-value > 0.05). Hence, a combined special cubic x linear model is appropriate to describe the observed data. The significant effects are the linear mixture of the three coagulants, the double interactions between *Opuntia robusta* biopolymer coagulant and doses (A × D), and *Uncaria tomentosa* biopolymer coagulant and doses (B × D). Therefore, the final equation in terms of components and factors for this response is described by Equation (3). A, B, and C are the proportions in the mixture of *Opuntia robusta* biopolymer coagulant, *Uncaria tomentosa* biopolymer coagulant, and aluminum sulfate, respectively, and D is coagulant doses.

(3) Chemical oxygen demand removal (%)=91.69×A+66.14×B+80.17×C−0.10×A×D−0.06×B×D

It can be noted that *Opuntia robusta* biopolymer coagulant affects the responses (turbidity and chemical oxygen demand removal) significantly more than *Uncaria tomentosa* biopolymer coagulant. The turbidity removal was not affected by *Uncaria tomentosa* biopolymer coagulant. Additionally, the chemical oxygen demand removal is more sensitive to the interaction between *Opuntia robusta* biopolymer coagulant and doses (*p*-value of 0.0094) than the interaction between *Uncaria tomentosa* biopolymer coagulant and doses (*p*-value of 0.0816) shown in Table 5. Therefore, *Opuntia robusta* has a higher potential as a biopolymer coagulant than *Uncaria tomentosa.*

**Figure 2:**
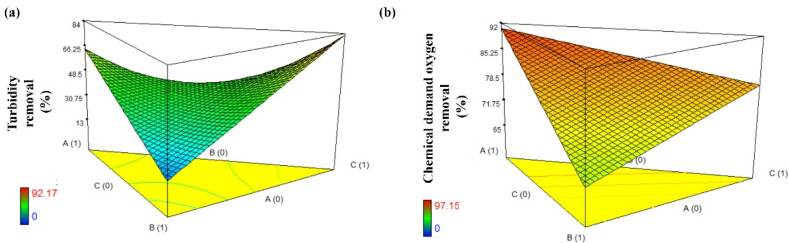
Three-dimensional response surface plots obtained from optimization analysis for the removal in urban wastewater of (**a**) turbidity and (**b**) chemical oxygen demand. A: *Opuntia robusta* biopolymer coagulant; B: *Uncaria tomentosa* biopolymer coagulant; C: Aluminum sulfate; D: Doses.

Since the objective of this work was to use the species *Uncaria tomentosa* and *Opuntia robusta* as biopolymer coagulants to reduce the consumption of chemical coagulants such as aluminum sulfate, an optimization analysis was performed using the following criteria: the maximum amount of the biopolymer coagulants, the minimum amount of aluminum sulfate, a dose range of 10 a 1000 mg/L, and Equations (2) and (3) as appropriate. The maximum percentages of removal, 70.5% and 90.1% for turbidity and chemical oxygen demand, respectively, are achieved using a dose of 10 mg/L of *Opuntia robusta* biopolymer coagulant. It can be observed in Figure 2a that the maximum efficiency of turbidity removal (84%) is achieved when aluminum sulfate is used alone. However, this efficiency decreases significantly when the aluminum sulfate is mixed with the *Uncaria tomentosa* biopolymer coagulant. However, the efficiency increases using *Opuntia robusta* biopolymer coagulant alone or in a mix with a proportion below 20% of aluminum sulfate. Figure 2b shows that the efficiency of chemical oxygen demand removal is decreased when *Opuntia robusta* biopolymer coagulant is mixed with *Uncaria tomentosa* biopolymer coagulant or aluminum sulfate. Therefore, the optimal dose of *Opuntia robusta* biopolymer coagulant is 10 mg/L.

A laboratory experiment was performed in triplicate under these optimal conditions and following the same methodology described in Section 2.3. Removal efficiencies of 68.7 ± 1.7% and 86.1 ± 1.4% for turbidity and chemical oxygen demand were obtained, respectively. Therefore, experimental errors of 2.5% and 4.4% were estimated regarding model prediction.

The increase in the electrical conductivity of the water caused by high coagulant doses was discussed in Section 3.2. However, it is assumed that this increase will not occur employing the optimal dosage of *Opuntia robusta* biopolymer coagulant (10 mg/L) since it is 20 times smaller than the lower limit of 200 mg/L used in the experimental design. Therefore, the application of *Opuntia robusta* biopolymer coagulant represents an alternative for reducing the use of aluminum sulfate (chemical coagulant) in urban wastewater treatment.

### 3.4. Effectiveness of the Biopolymer Coagulants

Table 6 shows a comparison between the effectiveness of biopolymer coagulants produced from *Opuntia robusta* and *Uncaria tomentosa* in this work and other biocoagulants reported by other authors.

Different biopolymer coagulants have been used for natural and synthetic water and industrial wastewater treatment. *Moringa Oleifera*, crustacean shells chitosan, tamarind seeds, banana pith, and *Jatropha curcas* seeds are the most efficient biocoagulants sources to remove turbidity in different water types (Table 6). However, *Opuntia robusta* biopolymer coagulant showed high efficiency in the chemical oxygen demand removal, even though this value is higher than the values reported by other authors (Table 6). Carpinteyro et al. [43] also studied *Opuntia mucilage* as a biocoagulant to treat cosmetic wastewater. *Opuntia* showed efficiency in turbidity and chemical oxygen demand removal. Therefore, *Opuntia robusta* biopolymer coagulant could represent an alternative for reducing the use of chemical coagulants in water and wastewater treatment.

On the other hand, *Uncaria tomentosa* biopolymer coagulant showed the lowest efficiency in removing turbidity. However, the efficiency of chemical oxygen demand removal is close to the values reported for tamarind seeds and *Opuntia* mucilage (Table 6). Hence, its application could be like a coagulant aid [44] mixed with aluminum sulfate under a dual coagulation system.

The cost of applying biocoagulants must be more competitive than using chemical coagulants. The cost of chemical coagulants for drinking water and wastewater treatment was estimated at USD 1.50 and USD 0.15–1.80/m^3^ of treated water. Additionally, the cost of biocoagulants was estimated at USD 0.0025-2 and USD 0.015–19.5/m^3^ of treated water. Although the application of some biocoagulants shows a higher price than chemical coagulants, the cost will be reduced when the cost of the resultant sludge handling is also included [6].

The cost of aluminum sulfate coagulant was estimated at USD 0.30–0.50/kg [45]. The production cost of the *Opuntia robusta* biopolymer coagulant was not calculated in this work since it could be subjective in estimating a value at laboratory level. Biopolymer coagulant was produced by extracting *Opuntia robusta* mucilage. Some authors have reported that this compound can be obtained from plants, fruits, seeds, and other sources locally available at a low cost [46]. Hence, this situation reflects that this biocoagulant could represent a lower-cost alternative material than chemical coagulants such as aluminum sulfate. Furthermore, the concepts of the circular economy could support the sustainability of using biocoagulants since the production and application of coagulants from natural sources bring on sustainable water treatment [4]. Therefore, it is necessary to perform a cost–benefit analysis and process standardization for the practical application of this biopolymer coagulant. *Opuntia robusta* biopolymer coagulant could represent a biodegradable, environmentally friendly, and low-cost material in water and wastewater treatment. The opportunities for its application in wastewater treatment at the industry level remain open.

## 4. Conclusions

This study evaluated the effectiveness of *Opuntia robusta* and *Uncaria tomentosa* biopolymer coagulants for removing pollutants from urban wastewater. Results showed that the coagulation treatment could be viable using *Opuntia robusta* biopolymer coagulant since turbidity and chemical oxygen demand removal efficiencies of 68.7 ± 1.7% and 86.1 ± 1.4%, respectively, were achieved at an optimal coagulant dose of 10 mg/L. These efficiencies were, respectively, 3.9 and 2.1 times higher than that using *Uncaria tomentosa* biopolymer coagulant at 200 mg/L. Therefore, the results support that *Opuntia robusta* mucilage is a promising alternative biopolymer coagulant. Additionally, it could be used as a green technology for treating wastewater and yielding value-added products.

## Figures and Tables

**Table 1:** Experimental design to evaluate the effectiveness of the biopolymer coagulants.

Trial Number	Components’ Proportion Values			Dose (mg/L)
	*Uncaria tomentosa* Biopolymer Coagulant	*Opuntia robusta* Biopolymer Coagulant	Aluminum Sulfate	
1	0.00	0.00	1.00	800
2	0.67	0.33	0.00	200
3	0.67	0.33	0.00	800
4	0.67	0.00	0.33	200
5	0.67	0.00	0.33	800
6	0.00	0.67	0.33	200
7	0.00	0.67	0.33	800
8	0.33	0.33	0.33	200
9	0.33	0.33	0.33	800
10	0.33	0.67	0.00	200
11	0.33	0.67	0.00	800
12	0.33	0.00	0.67	200
13	0.33	0.00	0.67	800
14	0.00	0.33	0.67	200
15	0.00	0.33	0.67	800
16	1.00	0.00	0.00	200
17	1.00	0.00	0.00	800
18	0.00	1.00	0.00	200
19	0.00	1.00	0.00	800
20	0.00	0.00	1.00	200
21	0.50	0.50	0.00	500
22	0.00	0.00	1.00	500
23	1.00	0.00	0.00	500
24	0.00	1.00	0.00	500
25	0.50	0.00	0.50	500
26	0.00	0.67	0.33	200
27	0.00	0.67	0.33	800
28	0.33	0.33	0.33	200
29	0.33	0.33	0.33	800
30	0.00	0.33	0.67	200

**Table 2:** Physicochemical parameters of the urban wastewater.

Parameter	Obtained Value	Reference Value	Unit
Temperature	10.00 ± 0.50	<25 ^a^	°C
pH	7.71 ± 0.02	6.5–8.5 ^a^	-
Electrical conductivity	1256 ± 1.00	150–500 ^b^	µS/cm
Turbidity	56.1 ± 0.50	<5 ^a^	NTU
Chemical oxygen demand	163.1 ± 2.00	60–150 ^c^	mg/L

^a^ [29], ^b^ [30], and ^c^ [31].

**Table 3:** Physicochemical parameters of water after coagulation treatment.

Trial Number	Doses (mg/L)	Change Values (Final−Initial)			Turbidity Removal (%)	Chemical Oxygen Demand Removal (%)
		Temperature (°C)	pH	Electrical Conductivity (µS)		
1	800	0.0	−1.2	+1030.5	88.5	87.2
2	200	−0.5	−0.1	+620.8	24.9	61.1
3	800	0.0	−0.3	+671.1	0.0	0.0
4	200	−0.5	−0.3	+691.6	58.9	72.7
5	800	−0.5	−0.7	+723.2	32.6	63.3
6	200	−0.5	−0.3	+691.6	39.5	67.1
7	800	0.0	−0.7	+712.1	88.1	83.0
8	200	0.0	−0.4	+694.4	42.5	69.7
9	800	0.0	−0.7	+754.9	17.7	51.2
10	200	0.0	−0.2	+632.0	38.9	66.3
11	800	0.0	−0.3	+661.8	1.6	34.5
12	200	+0.5	−0.3	+680.4	52.1	70.0
13	800	+0.5	−1.2	+591.0	0.0	0.0
14	200	0.0	−0.6	+696.8	67.6	72.7
15	800	0.0	−1.1	+916.9	20.0	58.4
16	200	−0.5	−0.2	+600.4	17.2	39.4
17	800	0.0	−0.6	+680.4	0.0	0.0
18	200	0.0	−0.1	+691.6	42.3	69.6
19	800	−0.5	−0.2	+658.1	0.00	0.0
20	200	0.0	−0.6	+704.6	97.2	97.2
21	500	0.0	−0.2	+650.6	28.3	62.9
22	500	0.0	−0.9	+916.9	88.6	92.6
23	500	0.0	−0.3	+669.3	15.2	37.4
24	500	−0.5	−0.2	+643.2	39.2	64.9
25	500	0.0	−0.6	+706.5	7.9	36.2
26	200	0.0	−0.3	+680.4	40.1	67.8
27	800	0.0	−0.7	+712.1	0.0	0.0
28	200	0.0	−0.4	+695.3	43.0	69.5
29	800	0.0	−0.8	+827.5	18.4	47.5
30	200	0.0	−0.6	+695.3	67.2	72.6

**Table 4:** Analysis of variance ANOVA for turbidity removal.

Source	Sum of Squares	Degree of Freedom	Mean Square	F Value	*p*-Value
Model	14,996.75	4	3749.19	9.70	<0.0001
Linear Mixture	9722.79	2	4861.39	12.58	0.0002
A × C	2207.85	1	2207.85	5.71	0.0247
A × D	3066.12	1	3066.12	7.93	0.0093
Residual	9661.22	25	386.45		
Lack of Fit	5781.05	20	289.05	0.37	0.9484
Pure Error	3880.17	5	776.03		
Cor Total	24,657.97	29			

A: *Opuntia robusta* biopolymer coagulant; B: *Uncaria tomentosa* biopolymer coagulant; C: Aluminum sulfate; D: Doses.

**Table 5:** Analysis of variance ANOVA for the chemical oxygen demand removal.

Source	Sum of Squares	Degree of Freedom	Mean Square	F Value	*p*-Value
Model	12,185.39	4	3046.35	6.48	0.0010
Linear Mixture	5200.90	2	2600.45	5.53	0.0103
A × D	3720.72	1	3720.72	7.91	0.0094
B × D	1548.06	1	1548.06	3.29	0.0816
Residual	11,756.12	25	470.24		
Lack of Fit	8307.86	20	415.39	0.60	0.8098
Pure Error	3448.27	5	689.65		
Cor Total	23,941.51	29			

A: *Opuntia robusta* biopolymer coagulant; B: *Uncaria tomentosa* biopolymer coagulant; C: Aluminum sulfate; D: Doses.

**Table 6:** Comparison between the effectiveness of biopolymer coagulants in the removal of pollutants.

Biopolymer Coagulant Source	Water Type	Doses Used (mg/L)	Effectiveness in the Removal of Pollutants	Reference
*Opuntia robusta* cladodes	Urban wastewater	10	68.7% of turbidity 86.1% of chemical oxygen demand	This study
*Uncaria tomentosa* leaves	Urban wastewater	200	17.2% of turbidity 39.4% of chemical oxygen demand	This study
*Moringa* *Oleifera*	Synthetic turbid Wastewater		82.43% of oil and grease	[38]
*Moringa* *Oleifera*	Drinking water	200	85% of turbidity	[12]
*Acacia mearnsii* tannin	Agricultural wastewater	5–8	70% of total phosphorous 82% of turbidity	[8]
*Solanum tuberosum* starch	Agricultural wastewater	1–2	80% of total phosphorous 82% of turbidity	[8]
Crustacean shells chitosan	Agricultural wastewater	5–10	95% of total phosphorous 98% of turbidity	[8]
*Plantago ovata* seeds	Turbid water	50	>80% of turbidity	[39]
Tamarind seeds	Detergent wastewater	400	97% of turbidity 39% of chemical oxygen demand	[40]
Banana pith	River water	100	98.5% of turbidity 54.3% of chemical oxygen demand 96.0% of suspended solids	[41]
*Jatropha curcas* seeds	Kaolin synthetic water	120	>96 of turbidity	[42]
*Opuntia* mucilage	Cosmetic Wastewater	150	50% of turbidity 38% of chemical oxygen demand	[43]

A: *Opuntia robusta* biopolymer coagulant; B: *Uncaria tomentosa* biopolymer coagulant, C: Aluminum sulfate; D: Doses.

## Data Availability

The datasets generated and/or analyzed during the current study are available from the corresponding author upon reasonable request.
